# Spatial and Time Domain Feature of ERP Speller System Extracted via Convolutional Neural Network

**DOI:** 10.1155/2018/6058065

**Published:** 2018-05-15

**Authors:** Jaehong Yoon, Jungnyun Lee, Mincheol Whang

**Affiliations:** ^1^Department of Biomedical Engineering, Duke University, Durham, NC 27708, USA; ^2^Department of Digital Media Engineering, Sangmyung University, Seoul 03016, Republic of Korea

## Abstract

Feature of event-related potential (ERP) has not been completely understood and illiteracy problem remains unsolved. To this end, P300 peak has been used as the feature of ERP in most brain–computer interface applications, but subjects who do not show such peak are common. Recent development of convolutional neural network provides a way to analyze spatial and temporal features of ERP. Here, we train the convolutional neural network with 2 convolutional layers whose feature maps represented spatial and temporal features of event-related potential. We have found that nonilliterate subjects' ERP show high correlation between occipital lobe and parietal lobe, whereas illiterate subjects only show correlation between neural activities from frontal lobe and central lobe. The nonilliterates showed peaks in P300, P500, and P700, whereas illiterates mostly showed peaks in around P700. P700 was strong in both subjects. We found that P700 peak may be the key feature of ERP as it appears in both illiterate and nonilliterate subjects.

## 1. Introduction

A brain–computer interface (BCI) is a system which provides a communication method by utilizing biophysiological signals [1]. BCI system enables the users to communicate with external world through measurements of biological signals and mostly do not require voluntary muscle movement. The system has been utilized to support severe locked-in syndrome (LIS) patients who lack motor ability, such as amyotrophic lateral sclerosis (ALS) and Guillain–Barre syndrome patients, as a means of communication [2–7]. Of many biophysiological signals, electroencephalography (EEG) has been most widely used in BCI field for its easiness in and low cost of measurement [8, 9].

Among different applications of BCI, event-related potential (ERP) based speller system has been one of the most widely used paradigms. The system was pioneered by Farwell and Donchin [10] in 1988 which utilized oddball paradigm in order to induce visual evoked potential (VEP), especially the P300 response. However, there are still illiteracy problems associated with ERP speller system [11, 12]. There has been reports of ERP features other than P300 [13, 14] which may be a key feature of distinguishing identifying illiterates.

One of the most prominent classification methods for ERP system is support vector machine (SVM) [15–18]. SVM is mathematically simple and, with sufficient knowledge of feature matrix, the experimenter can modulate the kernel for the target problem. Unfortunately, the kernel of SVM is sensitive to overfitting [19]. As EEG are measured from multiple electrodes [20–23], feature matrix can have high dimension with possible duplicates, which increase possibility of overfitting. As most of ERP system paradigms are dependent on P300 peak, the information (peak magnitude and latency) from each electrode should be similar. Moreover, it is hard to extract temporal and spatial information of EEG of a single kernel. Although multiple kernel learning (MKL) problem has been suggested [24], it is hard to extract intuition of the given problem through the method.

Recent development of deep learning provides an alternative approach. The convolutional neural network (CNN) can extract the feature from a given feature vector by using convolution. When an optimal filter is applied, the convolution will magnify the feature of interest and reduce the others [25]. CNN has been used in pattern recognition, especially in image recognition and speech recognition, as it provides topological information within the extracted feature [26–30]. Therefore, data with sequence or topological information can be recognized more efficiently as CNN enables extracting both temporal and spatial information within the raw data. As the ERP shows sequence of rise and fall as a response to visual stimuli, pattern recognition technique as CNN can be applied. Moreover, the convolution kernel of CNN can be used as tool for interpreting the spatial correlation among EEG electrodes.

In this paper, we explore the performance of CNN on ERP data to identify the key features that distinguish illiterates of ERP speller system. The convolution kernels of trained model will be explored to analyze the spatial correlation between cortices and pattern within ERP of each electrode. The subjects were grouped as either strong (nonilliterate) or weak (illiterate) depending on clarity of ERP signals. Results of two groups were compared to analyze difference in features.

## 2. Methods

### 2.1. ERP Speller Design

6 icons shown in Figure 2 were used as visual stimuli for the speller system of this paper. Rapid serial visual presentation (RSVP) panel design was adopted for the speller system to avoid gaze effect. During the experiment, screen size icons appeared on the center of the monitor in a random sequence [31]. The oddball paradigm was implemented by presenting target icon with distractors in a random sequence [10]. Each icon appeared 20 times per trial. The interstimulus interval (ISI) between icon appearances was set to 300 ms.

### 2.2. Data Acquisition

For this paper, 33 subjects (13 female, 20 male) participated in the experiment. The subjects' age ranged from 24 to 30 (mean = 27.25, std = ±1.92). During the experiment, subjects were asked to sit upright on a chair and instructed to keep still. No straps or ties were attached. Subjects were asked to self-report any inconvenience that might bother the concentration.

Each trial was initiated with an acoustic cue instructing the target of the given trial in subjects' mother tongue (Korean). 10 seconds after the acoustic cue was given, the icons appeared on the monitor according to RSVP design in random sequence. The subjects were instructed to mentally count the target occurrence during each trial (Figure 2(b)). Each session consisted of 12 trials. Each icon was selected as a target during the session twice in random sequence.

All subjects were naive; 10–20-minute preexperiment session was given to get subjects used to the procedure. The subjects were asked to self-report if they felt confident of the procedure. After the preexperiment session ended, the measurements of EEG were made. During the experiment, one training session and online session were conducted as a pair. To minimize subject's stress level and fatigue, 10-minute break was given in between training and online session. Each subject conducted minimum of 2 pairs of training and online session. No subjects had participated in more than 4 pairs of sessions.

EEG was collected by B-Alert X10 headset from Advanced Brain Monitoring (ABM) with sampling rate of 256 Hz. The EEG electrodes recorded followed international 10/20 system [32] as shown in Figure 2(a). All experiments were held in accordance with the Declaration of Helsinki, and the protocol was approved by the Ethics Committee of Sangmyung University.

### 2.3. Convolutional Neural Network

The architecture of CNN for this paper was as shown in Figure 2(c). The CNN consisted of 2 convolutional layers, 2 max-pooling layers, and 2 fully connected layers. Rectified linear unit (ReLU) function was applied as activation function for each convolutional layer since its performance was proven by another [33]. A softmax function was applied to output the last layer to regularize the final output to be between 0 and 1. The output of CNN was vector of 2 elements where each element represented the score of target and nontarget.

The CNN was designed to perform both spatial and temporal filtering. The feature maps of each layer were used to access correlation between adjacent electrodes and temporal feature of target ERP. In the 1st convolutional layer (L_1_), a filter of size 6 × 20 was applied to extract correlation of EEG recorded in adjacent electrodes. The row number of the filter was set to 6 as 3 electrodes were placed on each lobe (except for occipital lobe where two electrodes were placed). The size of filter enables analyzing the correlation of all 6 electrodes from adjacent lobes. For analysis of temporal feature of feature map from L_1_ among different lobes, a filter with size of 1 × 12 was applied for 2nd convolutional layer (L_2_) whose window size was approximately 100 ms in time scale.

To reduce the receptive field size for ease of calculation and prevent overfitting, max-pooling layers (M_1_ and M_2_) were inserted after each convolutional layer [27, 34]. The max-pooling layers downsample the feature map by applying a sliding window without overlap. As the name implies, the maximum value within the window is extracted. As the max-pooling introduces downsampling effect, a generalization of feature map was achieved which prevented overfitting of the model. Sliding window sizes of M_1_ and M_2_ were 2 × 2 and 1 × 10, respectively.

To further reduce the possibility of overfitting while training the model, drop-out technique was applied on the first fully connected layer (F_1_). The drop-out technique padded zeros to randomly selected rows in the given feature map. By intentionally losing the data within the feature map, generalization was achieved for the feature map which prevented the model from being overfitted by the training data [35, 36].

The size of input matrix fed into the CNN was 14 × 300 where each row corresponded to EEG collected from each electrode in Figure 2.

The CNN architecture was implemented in Python via TensorFlow on Python [37, 38]. The Adam optimizer was used to train the CNN which controls the learning rate to use larger step size. 10,000 iterations were conducted for training the model for each subject's data.

### 2.4. Tie Breaking

Ideally, if the model is perfect, only one icon will be identified as the target for a given trial. However, the system identified multiple icons as the targets in several trials. On the other extreme, the system failed to identify any target icon for some trials. For each case, the tie breaking rule was applied as follows.

- Multiple icons cases: When multiple icons were thought to be the target of a given trial by the CNN, the tie breaking rule was applied to select the target among these candidates. Since the first element of output vector represents the icons affiliation to target ERP property, the icon with the greatest value of the element was selected as the target of the trial.
- No target case: When the system failed to find the association of the ERP from any icons to property of target ERP, that is, no icons were identified as the target, same rules as those in multiple icons case were applied to select the target for the given trial. In this case, the first elemenet of output vector from all icons was compared. The icon whose first element of output vector was the greatest was selected as the target of the trial.

### 2.5. Analysis

Both qualitative and quantitative analysis were performed to analyze the characteristics of filters of each convolutional layer. The subjects were divided into two groups according to their relative strength of ERP as follows:

(i) ERP detection: if the target icon was detected as positive in a given trial, the ERP is considered detected. The subjects were divided accordingly into either H or L group (H and L for high and low) ERP detection group. The threshold between H and L group was 50%.
(ii) Feature map: feature maps from L_1_ and L_2_ were drawn in color map. As higher weights of feature map denote high discriminant power, the colormap can qualitatively give insight of how each electrode is correlated and at which time the main peak is formed.
(iii) Statistical analysis: for quantitative analysis of performance, accuracy, sensitivity, precision, F1 measure, and ROC were calculated for each subject and ANOVA test was held to compare mean difference. The accuracy is defined as the ratio of number of correctly identified trial to total trial numbers. The classical statistic measurements for quantitative evaluation are as follows:
  (1) TP≡true  positive,FP≡false  positiveTP≡true  negative,FP≡false  negativeSensitivity=TPFN+TPPrecision=TPTP+FPF1  measure=2×Sensitivity×PrecisionSensitivity+Precision.
(iv) Receiver operating characteristic: receiver operating characteristic (ROC), which plots the sensitivity against specificity, widely used statistical measurement for its diagnostic ability of binary classifier. As the CNN of the paper is a binary classifier, the ROC information is provided to compare the performance of CNN between H and L group.
(v) Peak signal to noise ratio: peak signal to noise ratio (PSNR) is used as measurement of qualitative reconstruction method of compression codes [39]. As the performance of filter will depend on how many core features are extracted from raw ERP, the PSNR of L_1_s feature map was calculated as a mean of measurement of performance. The greater PSNR shows the presence of significantly high weight inside feature map whereas lower PSNR represents only low weights that are present in the given feature map and the discriminant power of the filter is low.

## 3. Results

### 3.1. ERP Detection

Of 33 subjects, 19 were identified as H group. In Figure 3, time course of learning curve and other statistical measurements over the training iteration from H and L subject are presented. The learning curve of L subject shown in Figure 3(a) indicates that although the false negative rate (FN) drops according to the training iteration, reaching 0 eventually, the false positive rate (FP) becomes 1. Although the learning curve shows sharp increase at 1st and 13th iteration, mostly it remains around .2. This indicates that the CNN becomes overtrained to positives (target). Moreover, as the CNN identifies most of the ERP to be positive (high FP and low FN), the result indicates that discriminant feature of target ERP was not found. On the other hand, both FN and FP of H subject drop to around 0 and .2. The learning curve saturates around .85 indicating nonoverfitting of the CNN (Figure 3(b)).

The errors shown in Figures 3(c) and 3(d) are defined as follows for training and online data:

(2) $$\begin{array}{c} error=\frac{TP+TN}{TP+TN+FP+FN}. \end{array}$$

Although both H and L group show drop in both training and validation error as training iteration continues, the validation error of L subject is higher than that of H subject.

The ROCs of H and L subject shown in Figure 3(e) indicate the performance of CNN of H group to be greater than that of L group subject.

### 3.2. Spatial and Temporal Features

The feature map of each convolutional layer did not contain negative weights associated with negative peaks, such as N1 [40] as the activation function was set to ReLU [33].

The target ERP and feature map of L_1_ of sampler H and L subject are shown in Figures 4 and 6. The target ERP shown in both figures is target ERP averaged over all trials. To analyze the correlation of frontal and occipital lobe electrodes, the first 3 electrodes (first 3 rows of averaged target ERP matrix) were copied and pasted at the end of ERP matrix. As shown in Figure 4(a), the target ERP of L group subject shows broad peak around P700 range on F3 and CZ. ERP of other lobes did not show any significant positive weight indicating nonsignificant features associated with target being observed and being flat. Feature maps shown in Figures 4(b) through (i) have shown high correlation between ERP from central and parietal lobe electrodes.

On the other hand, the correlation of ERP among adjacent electrodes for H group subject shown in Figure 6 indicates the correlation is restricted to specific time range. Most of the high weights of feature maps shown in Figures 6(b), 6(d), 6(f), and 6(e) show significant positive value around P500 and P700 range for frontal and central lobe electrodes. The correlation between central and parietal lobe is shown in Figure 6(c) around P500 range. Some features around P500 region were found to show high correlation among all electrodes. Unlike that of L group subjects, feature map of L_1_ for H group subject showed high correlation among all electrodes, where each case shows specific temporal characteristics.

The temporal features shown in feature map in Figure 5 indicate that temporal features associated with P700 peak are present for L group subjects as expected. In Figures 5(a), 5(b), and 5(c), high positive weights were found around P700 range (row 4 and 6). However, most of the feature maps did not show significant weights or were either flat as in Figure 5(i).

The temporal features of H group subjects showed more variety. Some feature maps showed high positive weights in their feature maps around P300 and P500 range as shown in Figures 7(a), 7(b), 7(c), and 7(d), whereas the others indicated significant positive weight around P700 range as in Figures 7(a)–7(i). However, the weight associated with P700 range is more widely defined than those associated with P300 and P500.

### 3.3. Statistical Analysis

Comparison of classical statistical measurements and other measurements is shown in Table 1. The accuracy, sensitivity, and precision showed significant mean difference between H and L group (*p* values were 0.0135, 0.8.88e − 05, and 0.0072, resp.). A significant mean difference in F1 measure did not exist between H and L group. The accuracy of H and L group was 0.889 and 0.687, respectively. The sensitivity of H group was higher than that of L group, but the precision of H group was significantly lower than that of L group. The area under ROC of H group was significantly higher than that of L group (*p* value = 0.0137).

The PSNR for L_1_ of H group was significantly lower than that of L group. As all PSNR measured were negative, the absolute value of PSNR of H group was greater than that of L group. On the other hand, no mean difference of the peak time (PeT.) between H and L group was found (*p* value = 0.965).

## 4. Discussion

In this study, CNN has been used to investigate the spatial and temporal characteristics of ERP that distinguish the performance difference between illiterates and nonilliterates (L and H group). As a comparison of performance, classical statistic measurements as well as filter comparison measurement had been collected to compare the correlation of ERP taken from different EEG electrodes and identify characteristic temporal features associated with each group.

The statistical measurement shows that the mean performance of CNN with H and L group data had significant difference. The accuracy of H group data was higher than that of L group data. Interestingly, although the sensitivity of H group was higher than that of L group, the precision of H group was significantly lower than that of L group. This reflects the fact that the ERP of L group was not identified as target in most of the cases, and the CNN identified ERP from all 6 icons to be nontarget in more than half of the trials.

The learning curve and errors in Figure 3 demonstrate how the statistical measurement affects the performance of CNN. Although the false negative rate remains mostly near 0, as the false positive rate remains close to 0, the learning curve remains stable around .2 for the L group subject. This again reflects the characteristics of L group ERP who were mostly identified as nontarget. Some of the ERP that were identified as target ERP were mostly from nontarget icons, indicating lack of distinctive feature associated with target ERP. However, both false negative and false positive rate drop as training iteration continues for H group subject's data, leading to increase of learning accordingly to the iteration. As the ERP of L group does not have sufficient distinctive features, the model becomes slightly overtrained compared to the model of H group subject as shown in validation error plot in Figures 3(c) and 3(d). The comparison of ROC validates the analysis as ROC of H group was significantly higher than that of L group (*p* value = 0.0137).

As shown in Figure 4, most of the ERP collected from L group were flat in most of the channels. Most of the positive weights in target ERP were observed in frontal and central lobe electrodes (1st and 5th row of Figure 4(a)) which was contrary to the expectation as previous research indicated positive peaks associated with target event were mostly observed in parietal or occipital lobe [41, 42]. The correlation of ERP collected from adjacent electrodes did not show existence of significant correlation between occipital and parietal lobe data in L group subjects. On the other hand, ERP of H group were more invigorated, showing stronger activity in P300 area as shown in Figure 6(a). The ERP correlation indicated in feature map also indicated stronger correlation of ERP data collected from occipital and parietal lobe with other lobes. The spatial correlation shown in feature map of H group also indicated that the correlation was restricted in specific time range corresponding to either P300, P500, or P700.

**Figure 1:**
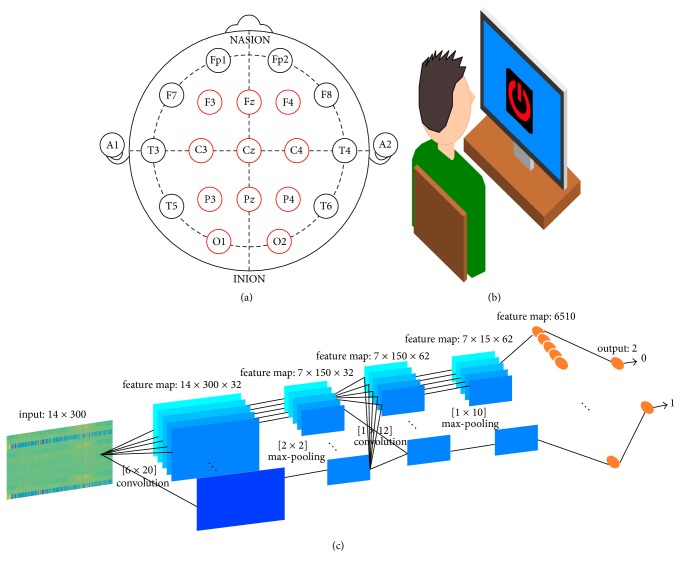
Experimental paradigm. (a) The position of EEG channels in 10/20 system. The EEG were collected from F3, F*z*, F4, C3, C*z*, C4, P3, P*z*, P4, O1, and O2 positions as indicated by red circle. (b) Experimental setting schematics. Subjects were sat on a chair and were asked to mentally count the occurrence of target icon. The ERP speller system for this paper was implemented with RSVP. The icon appeared on the center of the monitor. (c) Schematics of CNN architecture. The architecture consisted of 2 convolutional layers, 2 max-pool layers, and 2 fully connected layers. The number on top of each layer indicates size of feature map.

**Figure 2:**
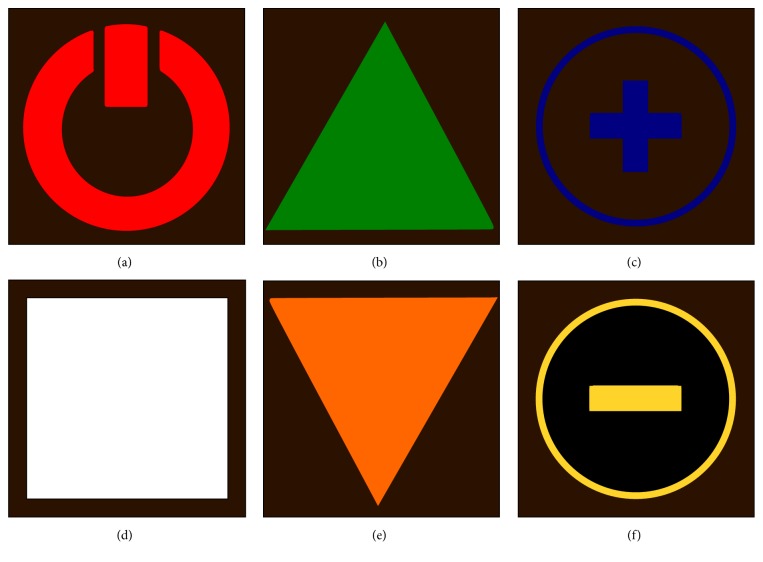
Schematics of icons used for rapid serial visual presentation (RSVP) panel. The design of icons was taken from television remote controller. (a) Turn on. (b) Volume up. (c) Channel up. (d) Turn off. (e) Volume down. (f) Channel down.

**Figure 3:**
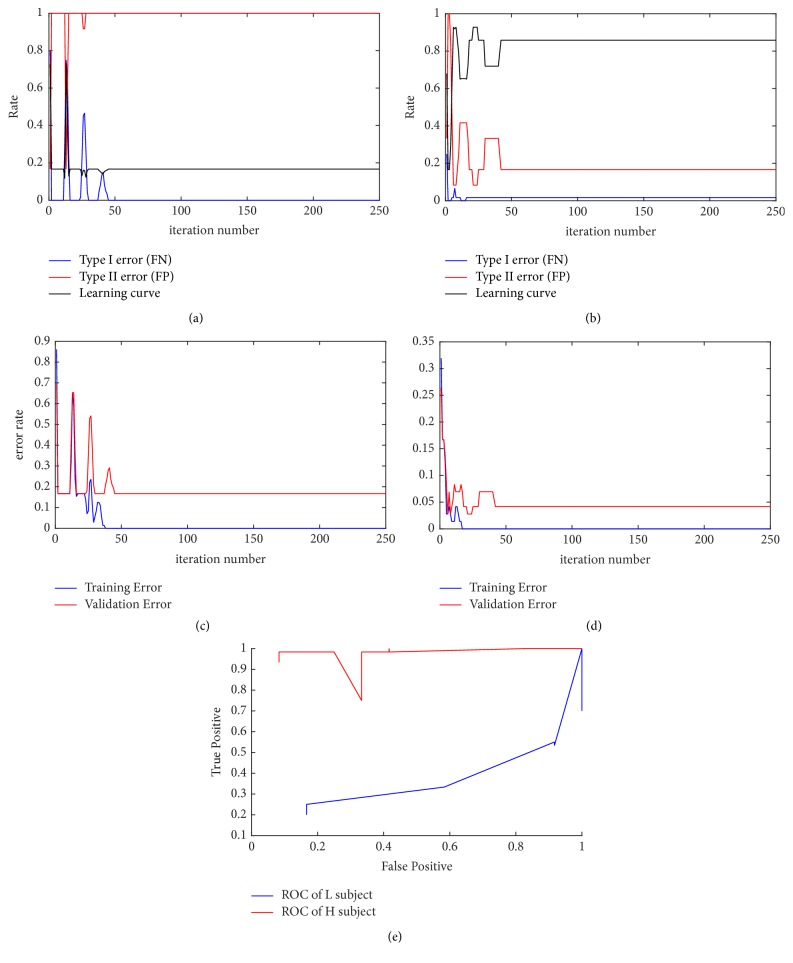
Learning curve and receiver operating characteristic curve (ROC) of L and H subject. (a) False negative rate (FN) and learning curve of L subject saturates near 0 and .2, respectively, whereas false positive rate (FP) increase to 1. (b) Both FP and FN drop over the time course for H subject and learning curve saturates near .8. (c) Training and validation error of drops over the time course for both L subject and (d) H subject. Both validation and training error are lower for H subject. (e) ROC curve of H and L subjects.

**Figure 4:**
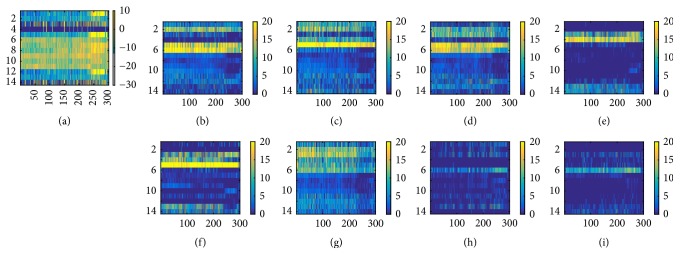
ERP averaged over all trials and feature map of L_1_ of L subject. The ERP from frontal lobe was copied and pasted on last three rows. (a) Grand average ERP over all trials. Feature maps from L_1_ shown in (b), (c), (d), (e), (f), (g), (h), and (i). Strong correlation between frontal and central lobe and between central and parietal lobe was found. Spatial correlation among other electrodes is not well defined.

**Figure 5:**
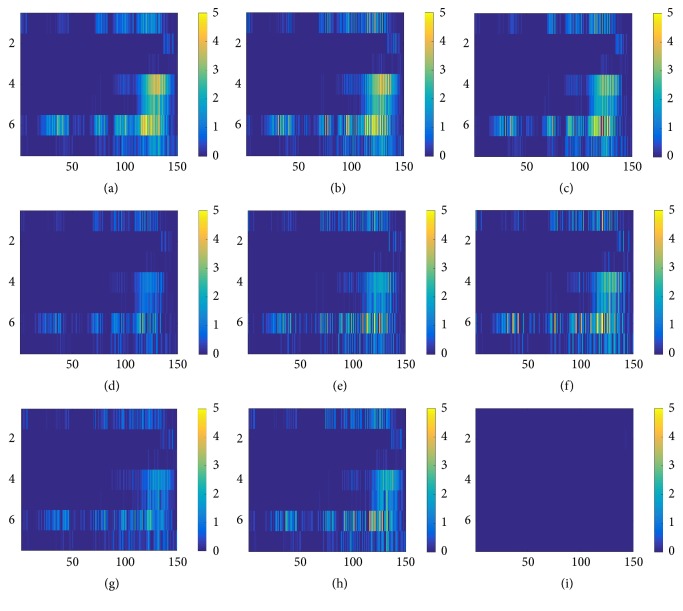
Feature map of L_2_ of L subject data. Temporal feature associated with P700 peak is found as shown in (a), (b), and (c).

**Figure 6:**
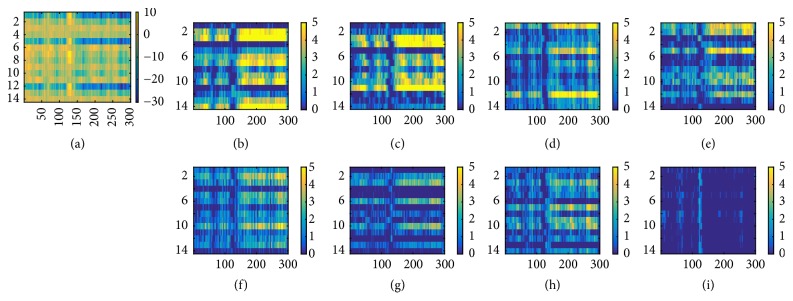
ERP averaged over all trials and feature map of L_1_ of H group. The format is the same as shown in Figure 4. (a) The grand averaged ERP of H group shows significant peak around P300 and P500 (rows 1, 5, 7, and 8). Correlation between ERP from adjacent electrodes shows high correlation related to specific time rage (P300 and P700) in (b), (d), (f), and (e).

**Figure 7:**
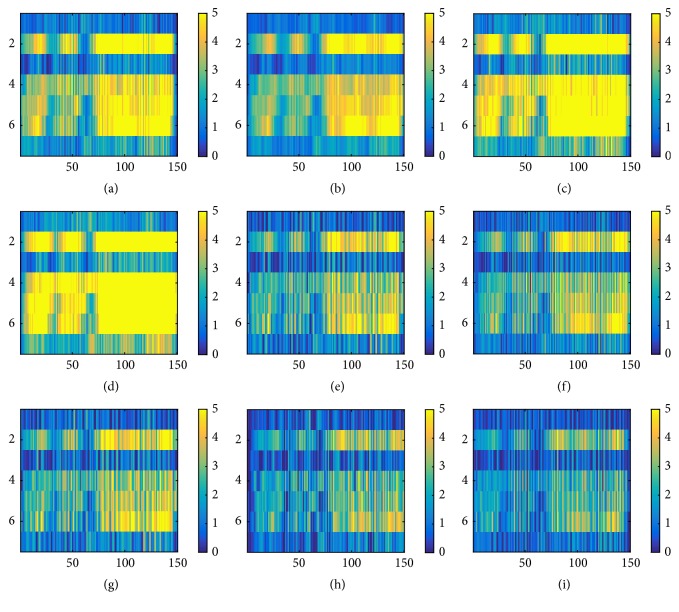
Feature map of L_2_ of H subject. Format is the same as Figure 5. High positive weight around P300 and P500 range were found in (a), (b), (c), and (d). (e)–(i) Moderate positive weight around P700 were also found.

The feature map of 2nd convolutional layer demonstrated the difference in temporal features between H and L group subjects. In most of L group subjects, the feature map did not show strong positive weights and was flat. Some indication of positive weights was mostly restricted in P700 region. On the other hand, the positive weights of H group were distributed around P300, P500, and P700 and the positive weights found near P300 and P500 range was sharper compared to those found around P700 range. Previous researches have indicated the possibility of existence of different features other than P300 [41, 43, 44] The result of the paper also supports the idea that P300 may not be the only key feaure of ERP speller system. Rather, the P700, which were identified among both L and H group subjects, may represent more universal ERP feature. However, the ERP from central lobe area observed in L group indicates the possibility of effect of stimulus probability [32] (Figure 1(a)).

The PSNR indicated that lack of activities in occipital/parietal lobe and broad peak found in P700 affect the performance of spatial filter in L1 as well. As the PSNR measures the maximum power of a signal and the power of corrupting noise [45], the result indicates that the filter was not able to extract distinctive signal of target ERP from background noise for L group subjects' data. This may be since peaks near P700 were broad and fluctuating. On the other hand, P300 and P500 peaks found in H group subjects were sharper, which made the filter extract relevant features more precisely without being affected by background noise. Interestingly, the major peak of L_2_ of H and L group subjects did not differ significantly (*p* value = 0.965). As the major peak was found by averaging the feature maps from L_2_, the difference in each feature map may have been overshadowed. Further statistical analysis to access temporal feature within each feature map must be applied to validate the results found in this study.

## 5. Conclusions

This study has investigated the difference in spatial and temporal features of ERP between high performance group (H group) and low performance group (L group). The result indicated that the major difference arises from spatial correlation of ERP among other lobes rather than temporal features. Although the temporal feature difference was not found to be quantitative in this study, the qualitative analysis indicated lack of P300 in low performance group. Interestingly, both low and high performance group showed activity near P700 which may be the key activity of ERP speller system instead of traditional P300 peak. Further analysis of individual feature map will be needed to investigate the key temporal feature of ERP speller system.

## Figures and Tables

**Table 1:** Results of the CNN classification. Data are sorted according to the ERP group. Accuracy (Acc.), sensitivity (Sens.), precision (Prec.), F1 measure, ROC, PSNR, and peak time of 2nd layer (PeT.) are given for comparison.

Subject number	Type	Acc.	Sens.	Prec.	F1 measure	ROC	PSNR	PeT.
1	H	.917	.250	.028	.050	.695	−42.285	.372
2	H	1.000	.647	.131	.218	.863	−34.468	.485
3	H	.917	.750	.188	.300	.997	−35.677	.594
4	H	.833	.750	.255	.344	.766	−32.909	.437
5	H	.833	.744	.242	.366	.660	−37.448	.354
6	H	.750	.782	.276	.408	.562	−39.263	.449
7	H	.833	.803	.292	.428	.814	−25.565	.595
8	H	1.000	.826	.317	.458	.873	−25.902	.411
9	H	.667	.844	.333	.478	.696	−22.070	.527
10	H	.750	.838	.346	.490	.873	−39.588	.367
11	H	.917	.869	.327	.475	.922	−25.750	.448
12	H	.917	.878	.342	.493	.940	−24.519	.664
13	H	.667	.747	.294	.422	.638	−23.987	.497
14	H	.833	.713	.279	.401	.778	−40.687	.543
15	H	.917	.721	.290	.414	.935	−39.207	.489
16	H	.750	.733	.302	.428	.998	−35.497	.362
17	H	.917	.733	.289	.415	.799	−38.910	.284
18	H	.917	.740	.295	.421	.861	−27.944	.452
19	H	1.000	.746	.298	.426	.780	−29.202	.458
20	L	.583	.846	.344	.489	.843	−25.722	.445
21	L	.583	.854	.343	.490	.573	−18.743	.575
22	L	.667	.849	.338	.483	.249	−21.219	.341
23	L	.833	.849	.341	.486	.427	−46.836	.638
24	L	.750	.853	.337	.483	.582	−20.236	.282
25	L	1.000	.860	.343	.488	.888	−20.511	.558
26	L	.917	.866	.343	.492	.535	−22.905	.627
27	L	.833	.868	.337	.485	.580	−22.883	.451
28	L	.750	.881	.362	.513	.898	−23.225	.381
29	L	.583	.808	.321	.460	.709	−31.783	.350
30	L	.833	.814	.324	.464	.874	−36.084	.396
31	L	.583	.755	.298	.428	.742	−27.483	.422
32	L	.667	.752	.303	.432	.931	−19.580	.533
33	L	.583	.745	.294	.432	.377	−32.561	.454
